# *Dendrobium officinale* Regulates Fatty Acid Metabolism to Ameliorate Liver Lipid Accumulation in NAFLD Mice

**DOI:** 10.1155/2021/6689727

**Published:** 2021-03-19

**Authors:** Shan-Shan Lei, Ning-Yu Zhang, Fu-Chen Zhou, Xinglishang He, Hui-Ying Wang, Lin-Zi Li, Xiang Zheng, Ying-Jie Dong, Rong Luo, Bo Li, Hai-Ying Jin, Qiao-Xian Yu, Gui-Yuan Lv, Su-Hong Chen

**Affiliations:** ^1^Collaborative Innovation Center of Yangtze River Delta Region Green Pharmaceuticals, No. 18, Chaowang Road, Xiacheng District, Zhejiang University of Technology, Hangzhou, Zhejiang 310014, China; ^2^College of Pharmaceutical Science, No. 548, Binwen Road, Binjiang District, Zhejiang Chinese Medical University, Hangzhou, Zhejiang 310014, China; ^3^Department of Medicine, Zhejiang Academy of Traditional Chinese Medicine, No. 132, Tian Mu Shan Road, Xihu District, Hangzhou, Zhejiang 310007, China; ^4^Zhejiang Senyu Co., Ltd., No. 8 Wanmao Road, Choujiang Street, Yiwu, Zhejiang 322099, China

## Abstract

*Dendrobium officinale* (DOF) is a traditional Chinese edible and officinal plant. Ultrafine DOF powder (DOFP) can regulate lipids and histopathology in the liver, but the underlying mechanisms of hepatic fatty acid (FA) metabolism, which is generally correlated with the development of nonalcoholic fatty liver disease (NAFLD), remain unclear. The purpose of the present study was to investigate whether DOFP treatment alters hepatic FA metabolism in NAFLD mice by using multidimensional mass spectrometry-based shotgun lipidomics (MDMS-SL) and analyse the underlying mechanisms. A 3-week DOFP treatment prevented lipid deposition and improved hepatic histopathology in NAFLD mice after withdrawal from the high-sucrose, high-fat (HSHF) diet, and it decreased triglyceride and FA content in the liver. Furthermore, the C16 : 0/C14 : 0 and C18 : 1/18 : 0 ratios in FAs were significantly decreased in the DOFP treatment group, and the C20 : 4/C20 : 3 and C22 : 4/C22 : 3 ratios were increased, and saturated FA was inhibited. Additionally, DOFP treatment significantly increased the content of two FA *β*-oxidation-related proteins (carnitine palmitoyltransferase 1-*α* and acyl-coenzyme A oxidase 1). It also decreased the content of a FA synthesis-related protein (fatty acid synthase), a FA desaturation-related protein (stearoyl-coenzyme A desaturase-1), and a FA uptake-related protein (fatty acid transport protein 2). Moreover, DOFP treatment improved dysregulated levels of major phospholipids in the livers of model mice. The results of this study confirm that DOFP treatment in NAFLD mice has liver recovery effects by regulating FA metabolism.

## 1. Introduction

Nonalcoholic fatty liver disease (NAFLD) is the most prevalent noncommunicable liver disease in the world, and it is the pathological manifestation of metabolic syndrome. It includes benign steatosis and severe nonalcoholic steatohepatitis (NASH), which is characterized by liver damage and inflammation, leading to cirrhosis and hepatocellular carcinoma [1]. NAFLD affects approximately 25% of the global adult population, placing a huge burden on human health [2]. Currently, there are no approved effective pharmacologic therapies for NAFLD [3, 4], and efforts to control NAFLD complications are far from satisfactory [5].

In NAFLD, 60% of the fatty acids (FAs) accumulated in the liver are derived from adipose tissue. Different FA sources, including dietary lipid intake, de novo adipogenesis, and adipolysis of adipose tissue, promote fatty liver development. FA metabolism disorders are highly correlated with the occurrence and development of NAFLD [6]. FA metabolism in the liver involves FA synthesis, uptake, desaturation, and oxidation, with related metabolic enzymes and proteins such as fatty acid transport protein 2 (FATP2) [7, 8], fatty acid synthase (FAS), stearoyl-coenzyme A desaturase-1 (SCD-1), acyl-coenzyme A oxidase 1 (ACOX-1) [6], and carnitine palmitoyltransferase 1 (CPT1) [9]. Recently, the roles of plant-based dietary supplements and traditional Chinese edible plants for NAFLD treatment via the regulation of FA metabolism in the liver have been studied extensively. For example, dietary supplementation with olive leaf extract and pu-erh tea has been shown to regulate FA metabolism to improve fatty liver in mice [6, 10].

*Dendrobii officinalis* (DOF) Caulis is the dried stems of the rare and endangered orchid *Dendrobium officinale* Kimura et Migo, of the family Orchidaceae. DOF, a natural edible plant, is one of the most important traditional Chinese medicines [11]. It is mainly distributed in Zhejiang, Yunnan, Guangxi, and other Chinese provinces. In China, it is prepared as a juice, powder, or soup. DOF can regulate blood lipids and protect liver function [12–15]. Our previous research has been devoted to the prevention and treatment of metabolic diseases with DOF [15, 16]. With modern cell wall-breaking technology, traditional Chinese medicines can be processed into micron-sized (1–100 *μ*m) ultrafine powder, such as ultrafine DOF powder (DOFP) resulting in convenient transport, easy storage, good water solubility, low functional component loss, and a high utilization rate, among other features beneficial to gastrointestinal and hepatic function [17, 18]. DOFP can ameliorate hepatic lipid lesions and reduce hepatic lipid accumulation in NAFLD mice [14]. However, the specific effect of DOFP on hepatic FAs remains under study, but it may be related to the effect of DOFP in NAFLD.

Thus, in this study, multidimensional mass spectrometry-based shotgun lipidomics (MDMS-SL) was used to evaluate the effects of a 3-week DOFP treatment on hepatic lipidomes in a NAFLD mouse model withdrawn from a HSHF diet. The effects were confirmed via hepatic lipid levels. Further investigations were carried out to determine whether metabolic enzymes involved in FA synthesis, desaturation, uptake, and oxidation in the liver were altered in response to DOFP treatment.

## 2. Materials and Methods

### 2.1. Chemicals and Reagents

Naringin and D (+)-glucose were purchased from Yuanye Biotechnology Co., Ltd. (Shanghai, China). Rutin was from Shi Feng Biotechnology Co., Ltd. (Shanghai, China). Total cholesterol (TC), triglyceride (TG), and hematoxylin-eosin (H&E) reagent were all purchased from Nanjing Jiancheng Technology Co., Ltd. (Jiangsu, China). CPT1-*α*, FATP2, SCD-1, ACOX-1, FAS, HRP conjugated goat anti-rabbit IgG (*H* + *L*), and GAPDH antibodies were all purchased from Proteintech Group (Chicago, USA). HRP conjugated goat anti-mouse/rabbit IgG (PV-6001) was from Zhongshan Goldenbridge Biotechnology Co., Ltd. (Beijing, China). BCA protein concentration determination kit, RIPA lysate kit, and chemiluminescent assay kit were from Beyotime Biotechnology. (Shanghai, China). All triheptadecenoyl glycerol (T17 : 1 TAG) was purchased from Nu Chek, Inc. (Elysian, USA). All other reagents were at least analytical grade and purchased from Fisher Scientific (Pittsburgh, USA), Sigma-Aldrich Chemical Company (St. Louis, USA), or as specified.

### 2.2. DOFP Preparation and Determination of Polysaccharides, Flavonoids, and Naringin of DOFP

**Figure 1:**
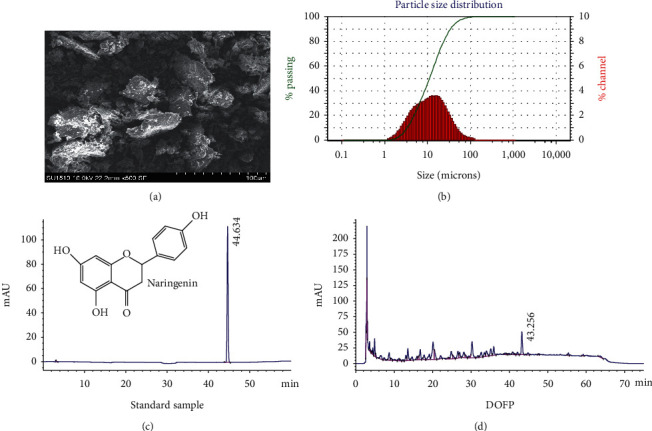
Preparation and characteristic of DOFP. (a) Representative photomicrograph of scanning electron microscope for DOFP. (b) Representative size distribution of DOFP particles. (c) High-performance liquid chromatography (HPLC) chromatogram of naringin standard. (d) HPLC chromatogram of DOFP.

The DOFP and the particle size distributions were measured as previously described [14]. After superfine grinding, critical changes occurred to the mean size of the DOFP particles, with *D*_90_ size distribution of 35.01 ± 1.19 *μ*m (Figures 1(a) and 1(b)). According to the methods from our previous study, the total polysaccharides in DOFP were determined to be 47.96% (*w/w*) according to the phenol-sulfuric acid method [14]. The total flavonoids were detected according to our previous literature [19], and the standard regression equation of rutin is *y* = 0.0106x − 0.009, *R*^2^ = 0.9989 (*y* was the absorbance, while *x* was the concentration of rutin). Rutin was used as reference standard for quantification.

Naringenin is one the most important kinds of flavonoids in DOFP, in this study we referred to the previous studied and measured the content by high-performance liquid chromatography (HPLC). HPLC was performed on an Agilent 2600 series HPLC system (1260, Agilent, Germany) consisting of quaternary pump, online degasser, well-plate autosampler, thermostatic column compartment, and diode-array detector (DAD). An LP-C18 column (4.6 × 250 mm, 5 *μ*m) was maintained at 30°C. Detection wavelength was set at 280 nm. The mobile phase consisted of methanol (A) and 0.2% phosphate solution (B) at a flow-rate of 1 ml/min. A gradient programme was as follows: 0∼5 min, 25%A; 5∼10 min, 25∼30%A; 10∼25 min, 30∼40%A; 25∼45 min, 40∼55%A; 45∼60 min, 55∼70%A; 60∼75 min, 70∼25%A; with a hold time of 75 min and injection volume 10 *μ*l. The standard regression equation of Naringenin is *y* = 35.259x − 19.209 (*R*^2^ > 0.9999).

### 2.3. Animal Study and Sample Collection

ICR mice (male, 18∼20 g, 4∼6 weeks, *n* = 28) were obtained from the Animal Supply Center of Shang Hai Si Lai Te (Shanghai, China). All the animals were raised in standard environmental conditions obeying the rules for the Use and Care of Laboratory Animals promulgate by the Zhejiang province in 2014. The animal operations were in accordance with the Guidelines of Care and Use of Laboratory Animals of Zhejiang University of Technology.

Firstly, the normal group (NG) (*n* = 9) was fed with regular diet, the model group (MG) mice (*n* = 19) were fed with high-sucrose, high-fat (HSHF) diet for 12 weeks, and one mouse of the NG and three mice of the MG were randomly sacrificed via euthanasia and collected liver tissues for hepatic pathology to confirm NAFLD, according our previous study. Then, the remaining eight mice were considered as the NG (*n* = 8), and the remaining 16 of the NAFLD model mice were randomly divided into two groups as the MG (*n* = 8) and DOFP administration group (DOFP) (*n* = 8) at a dosage of 0.6 g/kg per day for 3 weeks. At the same time, stop giving HSHF diet, replaced with regular feeding. The HSHF diet, with 20% sucrose, 15% lard, 1.2% cholesterol, 0.2% sodium cholate, and 63.6% standard diet, was obtained from Trophic Animal Feed High-Tech Co., Ltd. (Nantong, China).

At the end of experiment, mice were fasted overnight and blood was obtained from the ophthalmic venous plexus. The blood then was centrifuged at 3500 rpm for 10 min to obtain serum. Then, the mice were anesthetized and the liver was removed as soon as possible. According to our previous method [20], part of the liver was used for hepatic pathology, and other parts were used for hepatic lipids, free fatty acids (FFA), and western-blot analysis.

### 2.4. Histological Staining of Liver Section

Hematoxylin-eosin (H&E) and Oil Red O staining were performed according to our previous literature studies [14, 20, 21]. Oil Red O staining can be used to identify the lipid accumulation and quantify hepatic steatosis. Briefly, liver tissue was dehydrated with 30% sucrose solution before embedded into OCT compound (Tokyo, Japan) and cut into 10 *μ*m frozen sections for Oil Red O staining. Then, the cryosections were stained with 0.5% Oil Red O solution and the nuclei were counterstained with hematoxylin. All H&E and Oil Red O staining was photographed with the biological microscope (BX43, Olympus, Japan) and analysed with the Image-Pro Plus software.

### 2.5. Transmission Electron Microscopy (TEM)

The liver ultrastructure was observed by transmission electron microscopy (TEM) as we described previously [14, 20]. Briefly, the livers were cut into small pieces of 1 mm^3^, fixed in 2.5% glutaraldehyde phosphate buffer (pH 7.4) and then postfixed in 1% osmium tetroxide. And then the tissues were dehydrated in a graded ethanol series, embedding in epoxy resin for preparation of blocks. Ultrathin sections were cut into 50∼70 nm with ultrathin microtome. The ultrastructures of liver were observed with transmission electron microscopy (HT7700, HITACHI, Japan).

### 2.6. Measurement of Lipids in Liver and FFA in Liver and Serum

Liver tissue were put into ice cold saline and then homogenated to prepare 10% (*w/v*) homogenate suspension and centrifuged at 3500 rpm for 10 min, and the supernatant was used for determining the levels of TC, TG, and FFA in the liver. Meanwhile, the level of FFA in serum was also be detected. The measured methods were following the instructions of the kits.

### 2.7. Preparation of Lipid Extracts from Liver Samples and MS Analysis of Lipids

Triglyceride (TAG), FA, cardiolipin (CL), and phosphatidylcholine (PC) were identified and quantified by MDMS-SL; this method was described by Li et al. [20]. MS analysis of lipids and data processing were referred to the previous literature [22, 23].

### 2.8. Western-Blot Analysis of Proteins Involved in FA Metabolism

In brief, liver tissues were lysed at 4°C in the RIPA buffer. The tissue homogenates were centrifuged at 12,000 rpm for 15 min at 4°C, and the resulting supernatants were measured for total proteins by the BCA assay. Protein samples were separated with 10% SDS-PAGE, transferred onto a nitrocellulose membrane. Then, the membranes were blocked with 5% BSA for 2 h at room temperature and incubated with the corresponding primary antibodies (diluted GAPDH, FAS, CPT1-*α*, and FATP2) overnight at 4°C. After washed in PBST, the membranes were incubated with the appropriate HRP conjugated goat anti-rabbit secondary antibodies for 2 h at room temperature and washed again. The blotted protein bands were detected by chemiluminescent assay kit, and the protein expression levels were normalized to GAPDH by densitometry using the ImageJ software.

### 2.9. Immunohistochemistry (IHC) Analysis of FA Metabolism Dodes

The immunohistochemistry (IHC) staining was similar as we described previously [20, 24]. The expression and localization of SCD-1 and ACOX-1 in the liver were determined. In brief, the deparaffinized tissue sections were incubated with the corresponding primary antibody (1 : 200, dilution). A secondary antibody (HRP conjugated goat anti-mouse/rabbit IgG) was added. The signals were visualized by DAB staining, and the nuclei were counterstained with hematoxylin. Positive staining presented yellow color under microscope. The data of protein expressions were semiquantitative analysed as integrated option density (IOD) in positive area of the microphotograph with the Image-Pro Plus software.

### 2.10. Statistical Analysis

All data were expressed as the means ± SEM and subjected to one-way analysis of variance (ANOVA). The LSD *t*-tests were applied when homogeneity of variance assumptions was satisfied; otherwise, the Dunnett *t*-test was used. A value of *P* < 0.05 was considered to be statistically significant. Diagrams were performed by Graphpad Prism and Microsoft Excel software.

## 3. Results

### 3.1. Total Polysaccharides, Flavonoids, and Naringin in DOFP

Except for the total polysaccharides, flavonoids in DOF were main chemical constituents and important active substances. In our research, we followed by the methods described and the percentage content of total flavonoids was determined to be 3.78 ± 0.48%. Furthermore, the content of Naringenin was detected to be 0.3601 ± 0.014 mg/g (Figures 1(c) and 1(d)), which was one the most important kind of flavonoids in DOF.

### 3.2. DOFP Attenuated Lipid Levels in the Liver, but Not in the Serum

**Figure 2:**
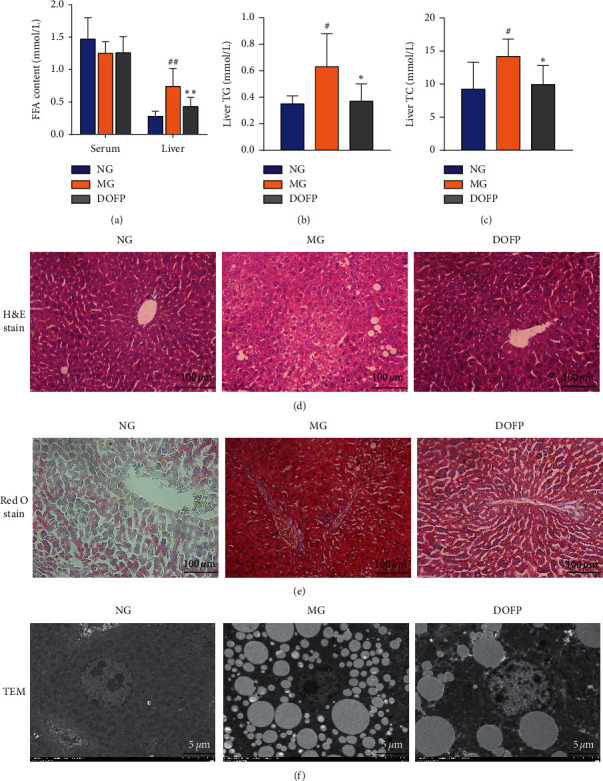
DOFP reduced lipids in the liver of mice with DOFP 3 weeks of intervention. (a) Concentrations of FFA in the serum and liver. (b) Concentrations of TG in the liver. (c) Concentrations of TC in the liver. (d∼f) H&E staining (400x), Oil Red O staining (400x), and TEM (1200x) of the liver. Significant differences are indicated by ^#^*P* < 0.05 and ^##^*P* < 0.01 as compared with the normal group (NG), and ^*∗*^*P* < 0.05 and ^*∗∗*^*P* < 0.01 as compared with the model group (MG) (*n* = 8 per group).

Liver FFA concentrations were significantly increased in the MG (*P* < 0.01). The increased FFA concentrations in the liver were robustly attenuated by DOFP treatment (*P* < 0.01). However, in serum, FFA concentrations in the DOFP group exhibited no significantly change compared with the MG (Figure 2(a)). Meanwhile, the levels of TC and TG in the liver showed the same tendency, being significantly increased in the MG and decreased in the DOFP group (*P* < 0.05) (Figures 2(b) and 2(c)). Furthermore, H&E and Oil Red staining of liver sections showed that the lipid droplets in the MG were increased in number and larger than those in the NG, and DOFP treatment improved this condition (Figures 2(d) and 2(e)).

Transmission electron microscopy (TEM) results showed that DOFP treatment could reverse lipid droplet accumulation and mitochondrial damage in the liver. Hepatocytes in mice fed a normal diet typically displayed large, round nuclei and no lipid droplets (Figure 2(f)). The ultrastructure of the livers in MG mice exhibited great lipid droplet accumulation and mitochondrial damage. The livers of mice treated with DOFP displayed little lipid droplet accumulation and mitochondrial damage. These results were consistent with the histological changes and fat content found in the livers. The results suggested that DOFP treatment could accelerate liver recovery by attenuating liver lipid levels in NAFLD mice.

### 3.3. DOFP Altered Different Triglycerides (TAGs) and FAs in the Liver

**Figure 3:**
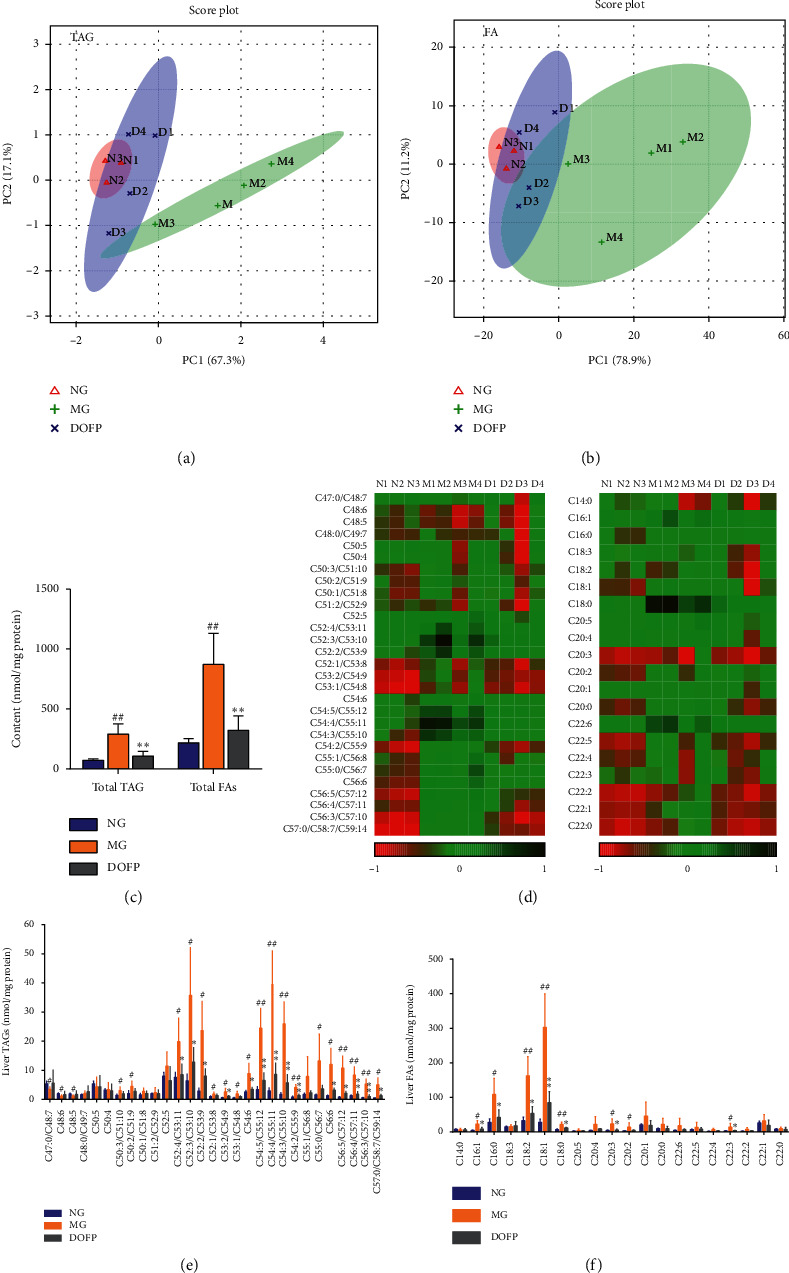
DOFP reversed TAG and FA metabolism abnormality in the liver of mice with DOFP 3 weeks of intervention. (a), (b) PCA of TAGs and FAs in the liver. (c) Total contents of TAGs and FAs in the liver. (d) Heat map of TAGs and FAs in the liver. (e), (f) Concentrations of differential TAGs and FAs in the liver. Significant differences are indicated by ^#^*P* < 0.05 and ^##^*P* < 0.01 as compared with the normal group (NG) and ^*∗*^*P* < 0.05 and ^*∗∗*^*P* < 0.01 as compared with the model group (MG) (*n* = 3∼4 per group).

There were 30 types of TAGs and 20 types of FAs specifically detected in the liver via the MDMS-SL method. The results showed that the total contents of TAGs and FAs in the MG were significantly increased but normalized in the liver via DOFP treatment (*P* < 0.01) (Figure 3(c)). Principal component analysis (PCA) describes the largest variation in data using a few orthogonal latent variables. Thus, PCA was carried out and the score plot was obtained for the TAG and FA data, including trends and groupings. For TAGs and FAs, PC1 separated the samples with respect to hepatic lipid levels and accounted for 67.3% and 78.9% of the total variance, respectively, whereas PC2 accounted for 17.1% and 11.2% of the total variance, respectively (Figures 3(a) and 3(b)). This indicated that the TAGs and FAs of hepatic lipid metabolism in the MG had worsened and that the administration of DOFP returned the levels toward normal values.

Further analysis of differential TAGs and FAs revealed that most types increased significantly and that DOFP could reverse this trend (Figures 3(d)–3(f)). These differential TAGs revealed that the C50 : 3/C51 : 10, C50 : 2/C51 : 9, C52 : 4/C53 : 11, C52 : 3/C53 : 10, C52 : 2/C53 : 9, C53 : 2/C54 : 9, C54 : 6, C54 : 5/C55 : 12, C54 : 4/C55 : 11, C54 : 3/C55 : 10, C54 : 2/C55 : 9, C55 : 0/C56 : 7, C56 : 6, C56 : 5/C57 : 12, C56 : 4/C57 : 11, C56 : 3/C57 : 10, and C57 : 0/C58 : 7/C59 : 14 values in the MG had a greater than two-fold change as when compared with those in the NG, while they were robustly ameliorated by DOFP treatment (*P* < 0.05,  0.01) (Figure 3(e)). The contents of C16 : 0, C16 : 1, C18 : 1, C18 : 2, C18 : 0, C20 : 2, C20 : 3, and C22 : 3 in the MG were significantly increased, and DOFP treatment significantly decreased the contents of C16 : 0, C16 : 1, C18 : 2, C18 : 1, C18 : 0, C20 : 3, and C22 : 3 (*P* < 0.05,  0.01) (Figure 3(f)). These data suggested that DOFP might reverse abnormal TAG and FA metabolism in the mouse liver.

### 3.4. DOFP Regulated FA Metabolism in the Liver

**Figure 4:**
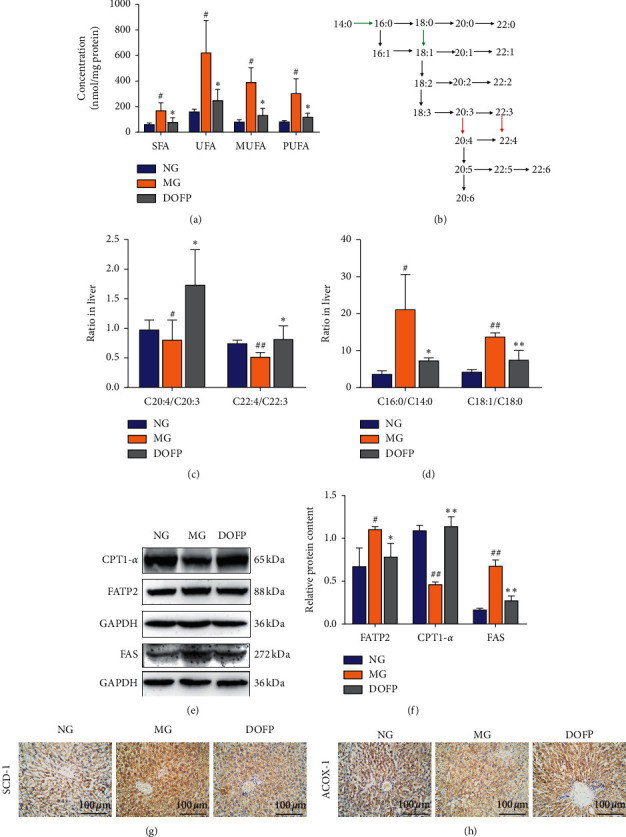
DOFP regulated FA metabolism in the liver of mice with DOFP 3 weeks of intervention. (a) FA species in the liver. (b) Biochemical pathway related to FA metabolism and ratios regulated by DOFP. Green arrows represent the ratios significantly elevated in the MG but attenuated by DOFP, while the red arrows represent the ratios significantly lowered in model group but elevated by DOFP. (c) Ratios of C20 : 4/C20 : 3 and C22 : 4/C22 : 3 in the liver. (d) Ratios of C16 : 0/C14 : 0 and C18 : 1/18 : 0 in the liver. (e) CPT1-*α*, FATP2, and FAS expression in the liver by western-blot analysis. (f) Quantitative analysis of FATP2, CPTI-*α*, and FAS protein levels. (g), (h) SCD-1 and ACOX-1 expression in the liver by IHC observed at magnification 400x. Significant differences are indicated by ^#^*P* < 0.05 and ^##^*P* < 0.01 as compared with the normal group (NG), and ^*∗*^*P* < 0.05 and ^*∗∗*^*P* < 0.01 as compared with the model group (MG) (*n* = 3∼4 per group).

The absolute concentration of differential FFAs in the liver showed that the levels of saturated fatty acids (SFA), unsaturated fatty acids (UFA), monounsaturated fatty acids (MUFA), and polyunsaturated fatty acids (PUFA) were all increased in the MG and they were all significantly attenuated by DOFP (*P* < 0.05) (Figure 4(a)). These differential FFAs are potential biomarkers, and they may be involved in altered enzymatic activity in FA metabolism.

To further investigate whether metabolic enzymes involved in the FA biosynthesis pathway were altered in response to DOFP intervention, 21 FA product/precursor ratios representing elongase and desaturase activity in FA synthesis were calculated (Figure 4(b)). As a result, two elongase ratios (C16 : 0/C14 : 0 and C18 : 1/C18 : 0) and two desaturase ratios (C20 : 4/C20 : 3 and C22 : 4/C22 : 3) were markedly altered in the livers of the MG and DOFP groups. The C16 : 0/C14 : 0 and C18 : 1/C18 : 0 ratios were significantly increased in the MG and decreased in the DOFP group, while the C20 : 4/C20 : 3 and C22 : 4/C22 : 3 ratios were decreased in the MG and increased in the DOFP group (P < 0.05, 0.01) (Figures 4(c) and 4(d)).

Furthermore, we measured the protein expression levels involved in FA synthesis, desaturation, uptake, and oxidation in the liver. FAS, which is a key enzyme for fatty acid synthesis, was concurrently increased in the MG and significantly decreased in the DOFP group (*P* < 0.01) (Figures 4(e) and 4(f)). Levels of SCD-1, which is a key rate-limiting enzyme in the desaturation of FFAs, were significantly elevated in the MG and attenuated in the DOFP group (Figure 4(g)). These results were consistent with the alterations of C16 : 0/C14 : 0, C18 : 1/C18 : 0, C20 : 4/C20 : 3, and C22 : 4/C22 : 3. Moreover, the protein related to hepatic FA uptake showed that the expression of FATP2 was concurrently increased in the MG and significantly decreased in the DOFP group (*P* < 0.05) (Figures 4(e) and 4(f)). CPT1-*α* and ACOX-1, which are related to hepatic *β*-oxidation, were concurrently decreased in the MG and significantly increased in the DOFP group (*P* < 0.01) (Figures 4(e), 4(f), and 4(h)). These results suggested that DOFP might reduce FA synthesis, desaturation, and uptake and promote FA *β*-oxidation, which may all be essential factors in regulating FA metabolism.

### 3.5. DOFP Altered Hepatic Phospholipid Levels

**Figure 5:**
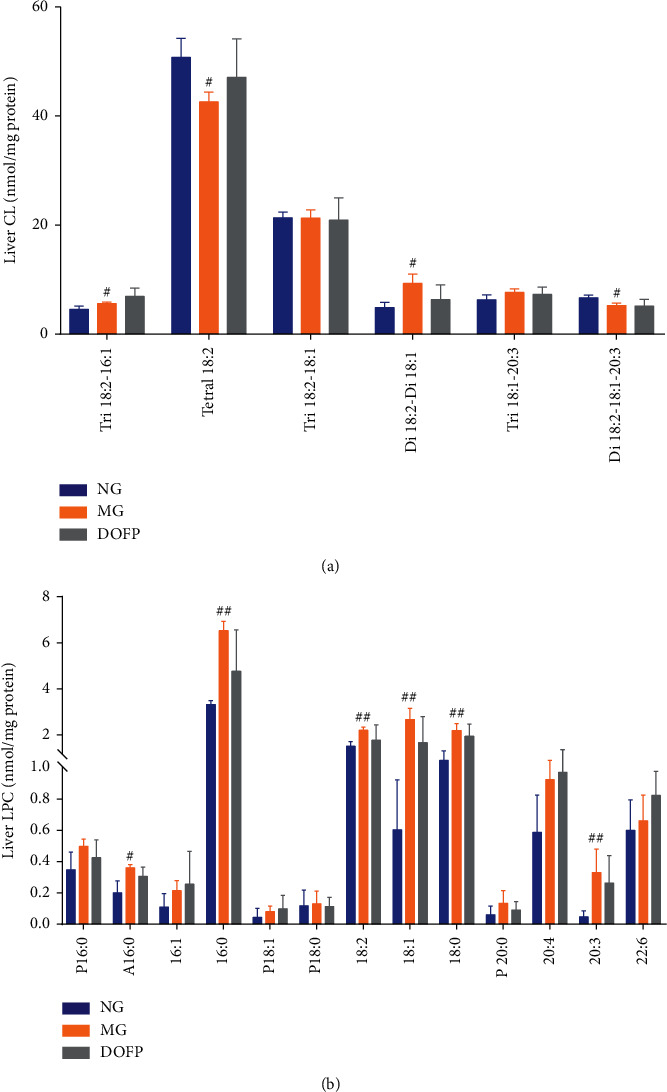
DOFP altered hepatic levels of phospholipid classes in the liver of mice with DOFP 3 weeks of intervention. (a) Cardiolipin (CL) species in the liver. (b) Lysophosphatidylcholine (LPC) species in the liver. Significant differences are indicated by ^#^*P* < 0.05 and ^##^*P* < 0.01 as compared with the normal group (NG), and ^*∗*^*P* < 0.05 and ^*∗∗*^*P* < 0.01 as compared with the model group (MG) (*n* = 3∼4 per group).

Cardiolipin (CL), which is a phospholipid containing four fatty acyl chains, is located almost exclusively in the inner mitochondrial membrane. The fatty acyl chains of CL are composed predominantly of linoleic acid and tetralinoleoyl CL (Tetra18 : 2), which accounts for more than 50% of total CL in most mammalian organs and is the most abundant CL species [22]. MDMS-SL analysis of the lipid extracts from the mouse livers demonstrated a significantly reduced percentage of Tetra18 : 2 in the MG (*P* < 0.05) (Figure 5(a)). Furthermore, Di18 : 2–18 : 1–20 : 3 was significantly decreased and Tri18 : 2–16 : 1 and Di18 : 2-Di18 : 1 were significantly increased in the MG compared with the NG (*P* < 0.05) (Figure 5(a)). DOFP regulated Tetra18 : 2, Tri18 : 2–16 : 1, Di18 : 2-Di18 : 1, and Di18 : 2–18 : 1–20 : 3 in the liver to normal levels, although this had no significant difference.

Phosphatidylcholines, which are a class of phospholipids, are a major component of eukaryotic cellular membranes. They play important roles as signaling molecule precursors and as key lipoprotein components [25]. Lysophosphatidylcholine (LPC) is a kind of phosphatidylcholine that is closely related to metabolic diseases, such as diabetes, atherosclerosis, dyslipidemia, and cardiovascular diseases [25]. MDMS-SL analysis demonstrated that a large number of LPC species, including A16 : 0, 16 : 0, 18 : 0, 18 : 1, 18 : 2, and 20 : 3, were significantly increased in the MG compared to the NG (*P* < 0.01,  0.05) (Figure 5(b)). DOFP treatment slightly corrected the increased mass levels of the LPC species in the liver. These data suggested that DOFP might alter the hepatic phospholipid levels, indicating that DOFP regulated FAs to ameliorate liver lipid accumulation in the NAFLD mice withdrawn from the HSHF diet for 3 weeks.

## 4. Discussion

Lipid accumulation in the human liver plays a key role in the pathogenesis and progression of NAFLD [1]. Hepatic lipid overload is a characteristic marker of NAFLD, which is associated with changes in hepatic lipid composition, and disturbed lipid homeostasis may play an important pathophysiological role in NAFLD. DOF is a traditional Chinese edible and officinal plant that can improve lipid metabolism in mouse models of NAFLD and liver injury [26, 27]. Furthermore, DOFP can accelerate liver recovery by regulating the gut-liver axis in NAFLD mice [14]. The results of the present study demonstrated that DOFP significantly reduced the levels of hepatic lipids, including TC, TG, and FFA, confirming that DOFP can improve liver lipid metabolism.

The ratio of SFA to UFA in the liver is a potential biomarker for chronic metabolic diseases, especially NAFLD [6]. In recent years, the relationship between hepatic FA metabolism and NAFLD has been investigated [6]. A 10-year follow-up clinical survey showed that the ratio of oleic acid to stearic acid (OA/SA, C18 : 1n9/C18 : 0) in the serum of nonhealthy obese patients with metabolic diseases was significantly increased, while the ratios of stearic acid to palmitic acid (SA/PA, C18 : 0/C16 : 0) and arachidonic acid to dihomo-*γ*-linolenic acid (AA/DGLA, C20 : 4n6/C20 : 3n6) were significantly decreased [28, 29]. The ratio of PUFA to SFA is an important biomarker for metabolic diseases [6, 30]. SFA can enhance the production of reactive oxygen species and proinflammatory factors and activate mitochondrial depolarization, leading to apoptosis [31]. Palmitate is more toxic than other FAs because it impairs insulin signaling in the liver, inducing liver cells apoptosis and impairing beta-cell response [32]. DOF can regulate lipids in ApoE^−/−^ mice [33] and in a hyperlipidemic model [34] associated with NAFLD. The present study revealed that DOFP could reduce the total amount of TAGs and FAs in the liver, further regulate the composition of important SFA, and promote hepatic recovery in mice with the fatty liver.

To further investigate the effect of DOFP on FA metabolism-related proteins in the liver, the levels of a FA synthesis protein (FAS), a FA uptake protein (FATP2), a FA desaturase protein (SCD-1), and two oxidative decomposition-related proteins (ACOX-1 and CPT1-*α*) in the liver were measured. FAS is a key enzyme for de novo lipogenesis, which plays an important role in the pathogenesis of NAFLD [35]. FAS expression was increased in NAFLD mice livers with lipid accumulation [36]. FATP2 is a member of the FATP family of FA uptake mediators. It has been independently identified as a very long-chain acyl-coenzyme A synthase of liver peroxisomes [7, 8]. FATP actively absorbs extracellular FFA; it also transforms intracellular FFA into lipoyl coenzyme A, reduces intracellular FFA concentrations, and promotes the transmembrane transport of FA from the blood to the liver [7, 8]. FATP2 expression in the liver is consistent with its primary role in FA transport and metabolism; when its expression is increased, there is a correlative increase in NAFLD progression [37]. Decreased FATP2 expression in the liver via shRNA reduces FA transport and protects mice from diet-induced NAFLD [7]. SCD-1, a key rate-limiting enzyme in FFA desaturation, is significantly elevated in the liver of obese mice on high-fat diets [6], and it is a potential therapeutic target for obesity, diabetes, and other metabolic diseases, including NAFLD [6, 38]. The results of the present study reveal that DOFP decreased FAS, FATP2 and SCD-1 expression in the liver of NAFLD mice, which was consistent with the increased MUFA content in model mice and the decrease of SFA and stearic acid with the DOFP use.

The mitochondria of hepatocytes can decompose FFA via *β*-oxidation, and the rate-limiting enzymes in this process mainly include CPTl-*α* and ACOX-l [6]. ACOX-1 is an enzyme related to FA oxidation in adipocytes, and it is an initiating enzyme of the *β*-oxidation system with peroxidase [39]. Studies have shown that a methionine-choline-deficient diet can attenuate ACOX-1 levels in the liver of NASH mice and that ACOX-1-deficient mice can develop severe NASH [40].

CPT1, which is a rate-limiting enzyme in FA *β*-oxidation, is located in the outer membrane of mitochondria, a key regulatory site for FA to enter mitochondria by transforming long-chain FAs from acyl-coenzyme A to carnitine [9]. In NAFLD mice, decreased CPT1 expression leads to the decreased transport of acyl-coenzyme A to mitochondria, thereby resulting in FA and acyl-coenzyme A deposition in the cytoplasm without entry into the mitochondria in the liver [10]. The results of the present study showed that DOFP could upregulate CPT1-*α* and ACOX-1 expression in the liver and accelerate FA decomposition. The results of MDMS-SL analysis also demonstrated that the total FA content in the liver was decreased. It is therefore suggested that improved FA metabolism in the liver via DOFP is related to increased FA oxidation.

*Dendrobium officinale* polysaccharide (DOP) is the main active ingredient of DOFP. According to our previous research, total polysaccharides account for 47.96% (w/w) of DOFP [14]. DOP protects the liver and ameliorates liver metabolism disorders. DOP has a protective effect on the liver, reducing abnormally increased ALT and AST levels [41, 42]. DOP treatment in a type II diabetes rat model was found to mitigate dysregulated levels of fatty acids, glycerolipids, glycerophospholipids, and ceramides, as well as disturbed bile acid metabolism [43]. Therefore, DOP may be the major compound responsible for the protective effect in NAFLD mice treated with DOFP.

Naringin is another major compound in DOFP. Naringin ameliorates high-fat diet-induced lipid metabolic disorder, hepatic dysfunction, and steatohepatitis [44, 45]. Jung previously reported that naringin regulated fatty acid metabolism to improve hyperlipidemia in a type II diabetes mouse model by significantly reducing the levels of plasma FFAs, plasma and liver triglycerides, liver carnitine palmitoyltransferase, and liver FAS [46]. Therefore, DOFP treatment in NAFLD mice may be related to the protective effect of naringin. All in all, we may further evaluate the effects of DOP and naringin on the regulation of fatty acid metabolism in future studies.

## 5. Conclusion

The results of the present study indicate that DOFP can ameliorate hepatic lipid accumulation in NAFLD mice via the regulation of FA metabolism in the liver by reducing FA synthesis, uptake, and desaturation and promoting FA *β*-oxidation. These results indicate that DOFP may be a potential therapeutic or interventional approach for NAFLD recovery.

## Data Availability

The data used to support the findings of this study are included within the article.
